# Surface Plasmon Enhanced Photocatalysis of Au/Pt-decorated TiO_2_ Nanopillar Arrays

**DOI:** 10.1038/srep26670

**Published:** 2016-05-24

**Authors:** Shuang Shuang, Ruitao Lv, Zheng Xie, Zhengjun Zhang

**Affiliations:** 1 State Key Laboratory of New Ceramics and Fine Processing, School of Materials Science and Engineering, Tsinghua University, Beijing 100084, China; 2 Key Laboratory of Advanced Materials (MOE), School of Materials Science and Engineering, Tsinghua University, Beijing 100084, China; 3 High-Tech Institute of Xi’an, Xi’an 710025, China

## Abstract

The low quantum yields and lack of visible light utilization hinder the practical application of TiO_2_ in high-performance photocatalysis. Herein, we present a design of TiO_2_ nanopillar arrays (NPAs) decorated with both Au and Pt nanoparticles (NPs) directly synthesized through successive ion layer adsorption and reaction (SILAR) at room temperature. Au/Pt NPs with sizes of ~4 nm are well-dispersed on the TiO_2_ NPAs as evidenced by electron microscopic analyses. The present design of Au/Pt co-decoration on the TiO_2_ NPAs shows much higher visible and ultraviolet (UV) light absorption response, which leads to remarkably enhanced photocatalytic activities on both the dye degradation and photoelectrochemical (PEC) performance. Its photocatalytic reaction efficiency is 21 and 13 times higher than that of pure TiO_2_ sample under UV-vis and visible light, respectively. This great enhancement can be attributed to the synergy of electron-sink function of Pt and surface plasmon resonance (SPR) of Au NPs, which significantly improves charge separation of photoexcited TiO_2_. Our studies demonstrate that through rational design of composite nanostructures one can harvest visible light through the SPR effect to enhance the photocatalytic activities initiated by UV-light, and thus realize more effectively utilization of the whole solar spectrum for energy conversion.

Semiconductor photocatalysis has been considered as an alternative for the degradation of different pollutants and demonstrated to be a technically viable cleanup process^1^. Among various oxide semiconductor photocatalysts, TiO_2_ has been intensively investigated because of its photostability, nontoxicity and low cost^2^. However, the low quantum yields and lack of visible light utilization hinder its practical application. So far, there are three main strategies to enhance the photocatalytic efficiency and visible light utilization of TiO_2_: (1) coupling with different semiconductors (e.g., TiO_2_/Cu_2_O^3^ and TiO_2_/WO_3_^4^), (2) combining with noble metals (e.g., Au/TiO_2_^5^, Pt/TiO_2_^6^ and Ag/TiO_2_^7^,^8^), and (3) introducing dopants (e.g., oxygen defects^9^, and sulfur^10^, nitrogen^11^). Among them, combining with metal are a promising method to develop highly efficient visible light photocatalyst. On the one hand, the deposition of the metal on TiO_2_ can greatly improve its photoefficiency through the Schottky barrier conduction band (CB) electron trapping and consequent longer electron-hole pair lifetime. Hu *et al*. reported a highly efficient Pt-doped TiO_2_ which have enhanced photocatalytic activity for NO_x_ oxidation both under UV and visible light irradiation^12^. The presence of Pt deposits on TiO_2_ is believed to retard the rapid charge-pair recombination by serving as an electron sink and facilitating interfacial electron transfer to dioxygen or other electron acceptors. Pt can also trap electrons on the conduction band, which are subsequently transferred to electron acceptors^13^. On the other hand, some noble metal nanoparticles (NPs) such as Ag and Au, exhibit strong UV-vis absorption due to their plasmon resonance, produced by the collective oscillations of surface electrons. Ingram *et al*. managed to reduce the high rate of charge-carrier recombination by combining a semiconductor photocatalyst with tailored plasmonic-metal nanostructures^14^. Pu *et al*. demonstrated those Au NPs, Au nanorods (NRs), and a mixture of Au NPs and NRs on the surface of TiO_2_ nanowire arrays could be prepared for effective photocatalysis and the activities were enhanced in both the UV and visible regions^15^.

As far as we know, there have been some research works of combining the plasmonic effect of Au and electron sink effect of Pt previously. Zhang *et al*. fabricated TiO_2_ electrospun nanofibers co-decorated with Au and Pt NPs into photocatalytic water reduction^16^. Atsuhiro *et al*. also synthesized TiO_2_ particles composited with Au and Pt NPs evaluated in the reduction of Cr^6+^ along with H_2_O oxidation^17^. Photocatalysts in previous reports were usually in the form of powders and in an amorphous state, which was hard to handle and restricted its practical applications. In the present work, we designed a plasmonic photocatalyst consisting of bimetallic Au/Pt-TiO_2_ supported on specific SiO_2_ substrates. Firstly, the vertically aligned TiO_2_ nanopillar arrays (NPAs) was fixed on specific SiO_2_ substrates by glancing angle deposition (GLAD) technique. Then, the Au and Pt NPs were deposited on TiO_2_ by using successive ion layer adsorption and reaction (SILAR) method^18^. Excellent photocatalytic property and stability were achieved and the fabrication of TiO_2_ self-standing structures, which will be easier for recycling and thus facilitate their potential applications in solar energy-driven photocatalysis.

## Results and Discussions

### Characterization of Photocatalysts

**Figure 1:**
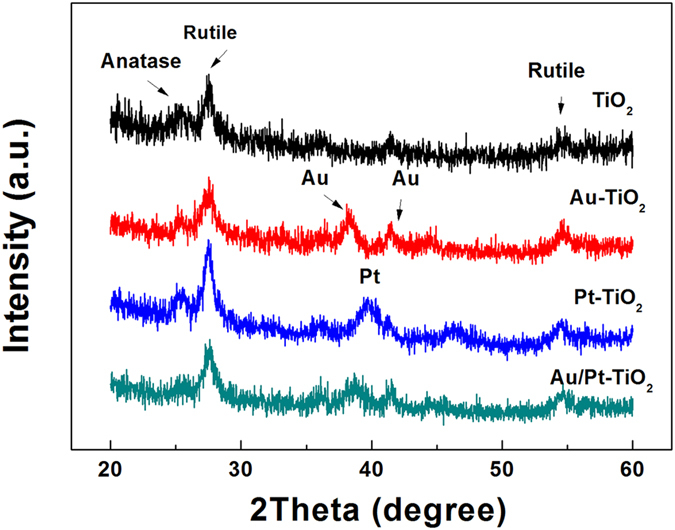
XRD patterns of the Au-TiO_2_ NPAs coated with 10 cycles, Pt-TiO_2_ NPAs coated with 10 cycles and Au/Pt-TiO_2_ NPAs.

Figure 1 presents the X-ray diffraction (XRD) patterns of different samples. All samples exhibit diffraction peaks at 25.2° and 27.3° corresponding to the (101) crystal planes of the anatase phase (JCPDS No. 21-1272) and (110) crystal planes of the rutile phase (JPCDS No. 21-1276). Beside this, the diffraction peaks (38.2°, 41.4°) assigned to Au (JCPDS No. 04-0784) and the peak at 39.7° assigned to Pt (JCPDS No. 04-0802) are displayed in Au-TiO_2_ and Pt-TiO_2_, respectively. These three peaks can be also observed in Au/Pt-TiO_2_ NPAs sample. Furthermore, XRD of Pt-TiO_2_ with different cycles is shown in Fig. S1 in supporting information. The peak intensity of Pt grows gradually as the deposition cycles increasing, indicating that more Pt NPs grows on the surface of TiO_2_ NPAs.

**Figure 2:**
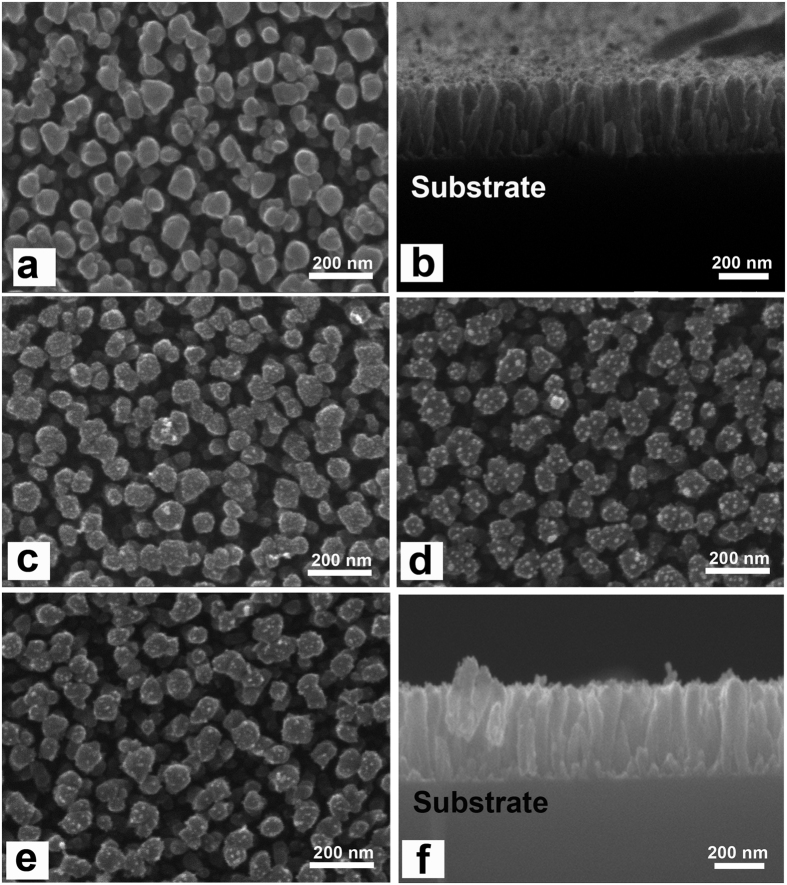
SEM images of the different samples: (**a)** TiO_2_ NPAs; (**b**) cross-section of TiO_2_ NPAs; (**c**) Pt-TiO_2_ NPAs coated with 10 cycles; (**d**) Au-TiO_2_ NPAs coated with 10 cycles; (**e**) Au/Pt-TiO_2_ NPAs; (**f**) cross-section of sample Au/Pt-TiO_2_ NPAs.

**Figure 3:**
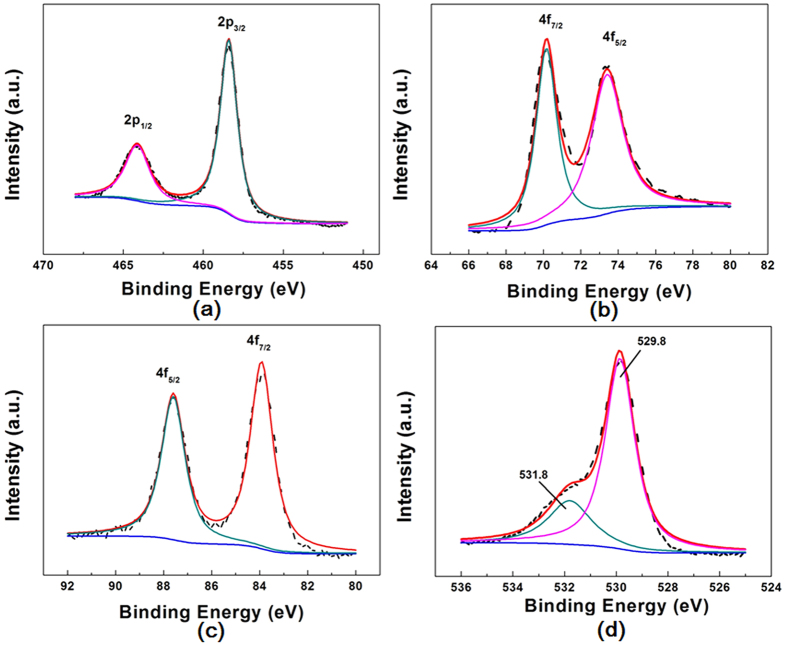
XPS spectra of Au/Pt-TiO_2_ NPAs coated with 10 cycles: (**a**) Ti 2p, (**b**) Pt 4f, (**c**) Au 4f, (**d**) O 1s.

Figure 2 shows the SEM images of the pure TiO_2_ film and those coated with Au, Pt and Au/Pt NPs with 10 cycles, respectively. As-annealed film consists of TiO_2_ NPAs with a diameter of ~50 nm and a length of ~200 nm (Fig. 2a). And it can be seen that these TiO_2_ NPAs vertically aligned on the Si substrate from sectional view (Fig. 2b). Au and Pt nanoparticles distribute uniformly on the TiO_2_ NPAs surface (Fig. 2c,d). The morphology of Pt-TiO_2_ with different cycles is also shown in Fig. S2. It is obvious to see that Pt nanoparticles appear as the bright spots and distribute uniformly on the TiO_2_ NPAs surface, and become more and more with increase of cycles. To affirm the state of the Pt and Au on the surface of TiO_2_, the Au/Pt-TiO_2_ NPAs sample was further characterized by XPS measurements (Fig. 3). The intense doublet of Au (83.8 and 87.4 eV) and Pt (70.4 and 73.5 eV) is due to metallic Au^0^, Pt^0^. These results confirm that the Au-Pt/TiO_2_ NPAs have been successfully fabricated on SiO_2_ substrates and the amount of Au or Pt can be adjusted by changing the SILAR cycle numbers.

**Figure 4:**
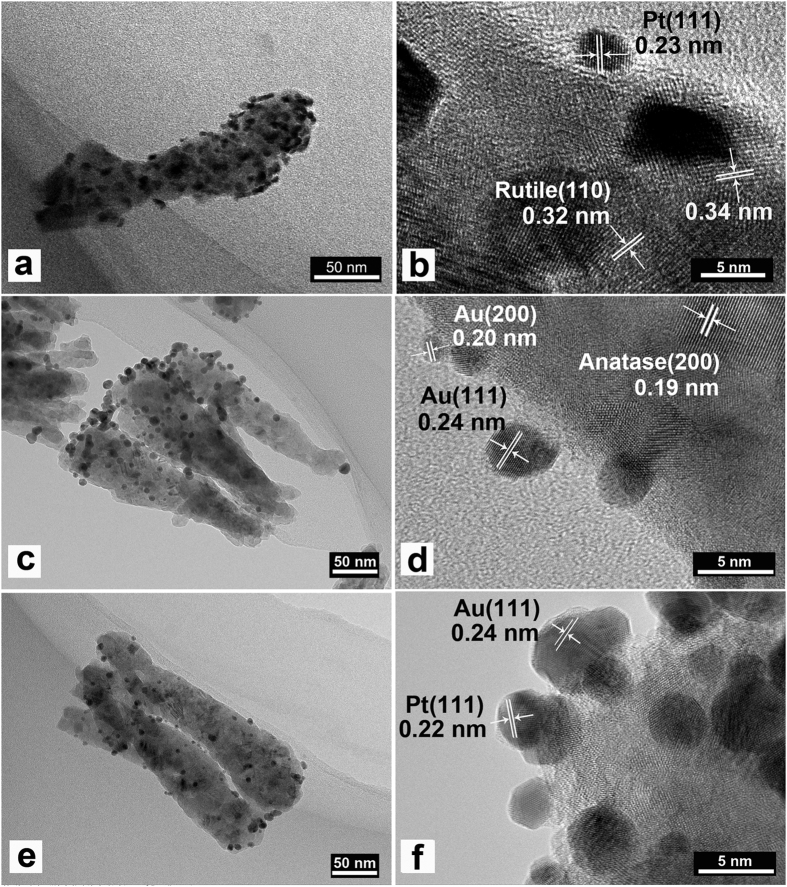
TEM images and HRTEM images of (**a,b**) Pt-TiO_2_ NPAs coated with 10 cycles; (**c,d**) Au-TiO_2_ NPAs coated with 10 cycles; (**e,f**) Au/Pt-TiO_2_ NPAs.

**Figure 5:**
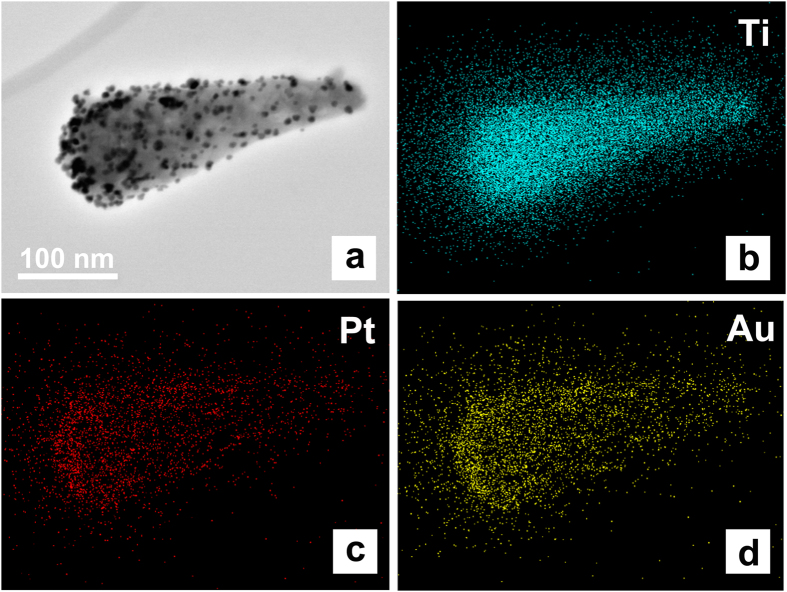
Morphology (**a**) and EDX elemental mapping of Au/Pt-TiO2 nanopillar arrays sample: (**b**) Ti; (**c**) Pt; (**d**) Au.

Furthermore, TEM images in Fig. 4 shows that Au and Pt NPs are uniformly dispersed on the surface of TiO_2_. Their average sizes are about ~4 nm, and in a regular cubic shape. According to the measurement of lattice fringes, d = 0.23 nm, 0.24 nm, 0.34 nm and 0.32 nm match very well with the crystallographic planes of Pt (111), Au (111), anatase (101) and rutile (110), respectively. This result indicates that Au, Pt and TiO_2_ are effectively interfaced. The formation of metal-semiconductor nanojunctions, including Au-TiO_2_ and Pt-TiO_2_, could be favourable for interfacial charge transfer among the three components, enhancing photocatalytic activities of the composites. In addition, the existence of anatase–rutile heterojunction in the NPs may help the rutile particles to efficiently collect photon-induced electrons from the anatase particles to reduce the carrier recombination^19^. Furthermore, the Ti, O, Au and Pt elemental mapping of Au/Pt loaded TiO_2_ nanopillars was also characterized in Fig. 5 which shows both morphology images and elemental maps of samples. It can be seen that metal particles concentrate more on the one end of nanopillar. And from element analysis, Au and Pt element are spatially homogeneous corresponding with noble metal particles distribution.

Diffuse reflectance UV-vis spectra of three typical resultants (Au-TiO_2_ NPAs, Pt-TiO_2_ NPAs coated with 10 cycles and Au/Pt-TiO_2_ NPAs) are shown in Fig. S3, which are converted into Kubelka-Munk function. TiO_2_ exhibits a UV absorption band around 200~400 nm. The Au-TiO_2_ NPAs show obvious enhancement on light absorption in the visible region with a broad band centered at around 540 nm assigned to the surface plasmon absorption of embedded Au NPs^20^,^21^,^22^. However, the SPR absorption band is hardly observed in the spectrum of Pt-TiO_2_ NPAs because of the low imaginary part of the dielectric function of Pt.

### Photodegradation of MO

**Figure 6:**
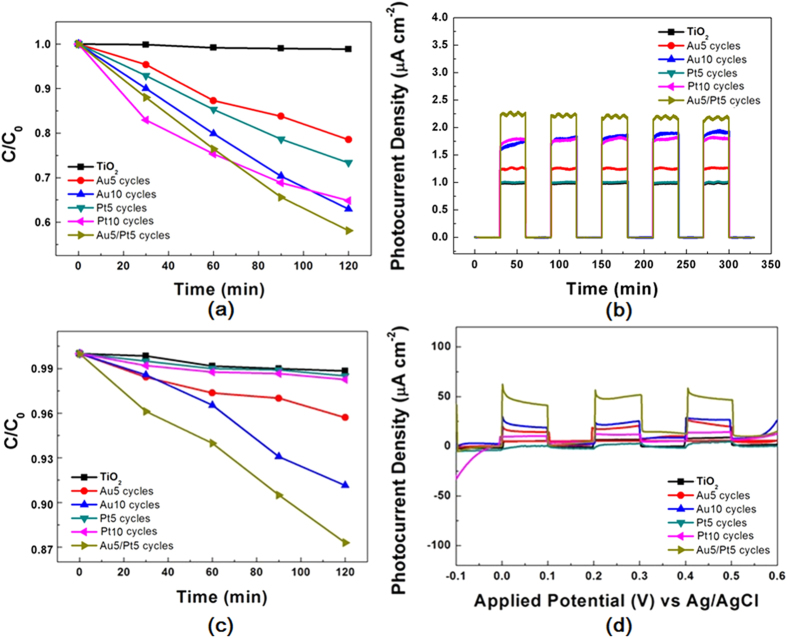
UV-vis light degradation of (**a**) MO and (**b**) current versus time measurements; visible light (λ ⩾ 420 nm) degradation of (**c**) MO and (**d**) current versus applied potential vs Ag/AgCl measurements.

To evaluate the effect of bimetal Au-Pt on the photocatalytic activity of TiO_2_, the photodegradation of MO was carried out under visible irradiation. As a comparison, MO degradation were also performed in Pt/TiO_2_ NPAs, Au/TiO_2_ NPAs and TiO_2_ NPAs. As shown in Fig. 6, neither TiO_2_ NPAs nor Pt-TiO_2_ NPAs show any activity for the MO degradation, while MO is evidently degraded by Au-TiO_2_ NPAs after 120 illumination under visible lights. Under UV-vis lights, mix Au/Pt-TiO_2_ sample shows better photocatalytic performance than single metal decoration sample no matter dye decomposition or photoelectrical test (Fig. 6a,b). In the range of wavelength λ > 420 nm, only Au NPs have light absorption, and the degradation of MO in Au-TiO_2_ NPAs is from the plasmon-induced Au NPs. Moreover, the rate of MO photodegradation on Au/Pt-TiO_2_ NPAs is 1.36 times faster than that on Au-TiO_2_ NPAs. Therefore, Pt NPs also plays an important role in the enhanced activity of Au/Pt-TiO_2_ NPAs. Fig. S4(a) presents the photodegradation of MO by series Pt-TiO_2_ catalysts under simulated solar irradiation. In absence of Pt NPs, the MO is degraded by TiO_2_ only 6.5% until 120 min. While the enhancement is observed after the introduction of Pt NPs. The amount of Pt on the surface of TiO_2_ relates with the SILAR cycles, and then effects the photocatalytic activities. With the loading amount of Pt increasing, the degradation rate of MO increases and reaches maximum at 10 cycles, then decreases at 15 cycles. The results indicate that reasonable amount of Pt could enhance the overall photocatalytic efficiency in contact with TiO_2_.

Figure S4(b) compares the photocurrent density of series Pt-TiO_2_ NPAs with light on/off under simulated solar irradiation at a bias potential of −0.1~+0.6 V. Obviously, Pt-TiO_2_ NPAs exhibit a much higher photocurrent than TiO_2_ NPAs, indicating that more separation of photogenerated carriers occurred in the former. The photocurrent of Pt-TiO_2_ NPAs reaches to maximum at 10 cycles (i.e. 2.5 mA·cm^−2^ *vs* +0.5 V) and then decreases at 15 cycles, suggesting the electron sink effect is depended on the dispersion of Pt. The Pt NPs in the samples at 15 cycles are aggregated, becoming the recombination center of electron-hole. Therefore, reasonable amount of Pt NPs in Pt-TiO_2_ NPAs composite could act as a sinker for photoinduced charge carriers, promoting charge separation to enhance the overall photocatalytic efficiency in contact with TiO_2_. Similar phenomenon has been observed in Au-Pt/TiO_2_ system under visible light irradiation (Fig. 6d). Compared with the photocurrent of Au-TiO_2_, which of Au-Pt/TiO_2_ NPAs are remarkably enhanced, indicating that the later sample exhibit higher charge separation efficiency. To evaluate the stability of the Au-Pt/TiO_2_ NPs, recycling test was performed on the degradation activity under both UV-vis and visible lights. Figure S5 displays the MO degradation performance in a cycling photocatalytic run under same condition with previous. After five recycles, degradation efficiency does not observably decline, indicating the good stability of the catalyst during the photocatalytic reaction.

PL technique is also an effective way to study the efficiency of the charge carrier trapping, migration and transfer, as PL signals result from the recombination of photo-induced carriers. Figure S6 shows the PL spectra of the TiO_2_ NPAs, Au-TiO_2_ NPAs, Pt-TiO_2_ NPAs, and Au/Pt-TiO_2_ NPAs. The peaks at ~425, ~530 nm can be ascribed to the self-trapped excitons and the oxygen vacancies (V_o_) in TiO_2._ After decorated by metallic particles, these PL peaks are weaker. The lower PL intensity of Au/Pt-TiO_2_ NPAs suggests a lower recombination rate of the photo-induced electron–hole pairs, resulting a better photocatalytic performance. While PL intense of Au-TiO_2_ NPAs and Pt-TiO_2_ NPAs are between TiO_2_ NPAs and Au/Pt-TiO_2_ NPAs which corresponding with dye degration results.

### Photocatalytic Mechanism

**Figure 7:**
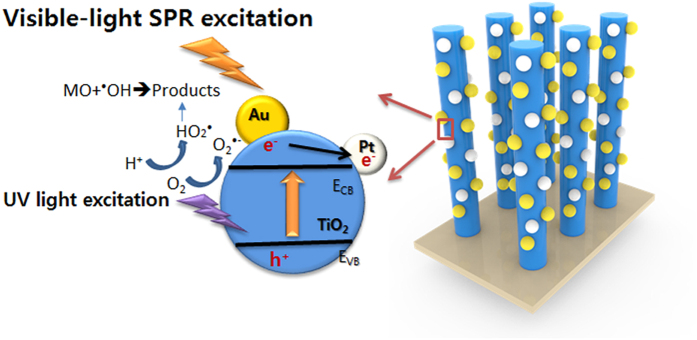
The photocatalytic process for Au/Pt-TiO_2_ NPAs under UV-vis lights.

Under visible irradiation, the incident photons are absorbed by Au NPs (SPR peak wavelength: ~520 nm in visible light region) through SPR excitation^23^. Then, electrons transfer from the plasmon-excited Au to the conduction band of TiO_2_^24^. Since the work function of Pt metal (5.40 eV from vacuum^25^) is larger than that of Au metal (4.78 eV from vacuum), that is, the Fermi level of Pt is lower than that of Au, electron transfer from Au to Pt through TiO_2_ is reasonable. Pt NPs act as cocatalyst at which electrons can either reduce the dye or can react with electron acceptors (O_2_ absorbed on the surface of Ti^3+^ or dissolved in water) to create superoxide radicals (O_2_^•−^). Meanwhile, the resultant electron-deficient Au particles can oxidize the organic molecule or react with OH^−^ to form hydroxyl radicals, OH•, which are highly oxidizing species. The process is shown in Fig. 7. And co-decoration of Au/Pt not only expand TiO_2_ to visible light region, but also increase the efficiency of charge separation, improving its photocatalytic efficiency. In addition, it has also been verified that active radicals are also produced from UV photoexcited TiO_2_ creating electron-hole pairs to react with adsorbed oxygen/H_2_O^26^. Therefore, Under UV irradiation, the highly efficient degradation of dyes comes from both photoexcited TiO_2_ and plasmon-excited Au NPs.

## Conclusion

In summary, we have successfully fabricated Au/Pt NPs-decorated TiO_2_ composite NPAs by using SILAR technique. As compared to Pt-TiO_2_ and Au-TiO_2_ NPAs it exhibits remarkably improved photocatalytic activities for not only degradation of dye but also PEC performance. These enhanced photocatalytic activities through codecoration of Au and Pt NPs are attributed to the synergy of electron-sink function of Pt NPs and Au SPR effect that improves charge separation of photoexcited TiO_2_. Our studies demonstrate that through rational design of composite nanostructures one can utilize a high-energy photon in the solar spectrum to generate charge carriers for photocatalytic reactions. Meanwhile, visible light in the solar spectrum can be synergically used for SPR excitation to enhance the charge separation and photocatalytic efficiency as well. This provides a more effective way to harvest solar energy for decomposition reaction.

## Methods

### Synthesis of TiO_2_ NPAs

Vertically aligned Ti NPs were deposited by the e-beam GLAD technique onto three different substrates as below: (1) planar silicon substrates with (001) orientation for material characterization, (2) quartz substrates for degradation reaction, and (3) F-doped SnO_2_ (FTO) substrates (20 Ω per square) for photoelectrochemical (PEC) performance test. All the substrates were ultrasonically cleaned in acetone, ethanol and deionized (DI) water baths in sequence for 10 min, respectively. Prior to the deposition, the chamber was evacuated to a vacuum level above 1 × 10^−8^ Torr. During deposition, the vapor flux incident angle was set to ~86° off the surface normal to the substrates, rotating at a speed of 10 rpm. The deposition rate (~0.75 nm•s^−1^) and the height of the NPs were monitored by a quartz crystal microbalance. After which the Ti films were oxidized in a tube furnace in order to obtain TiO_2_ NPAs. The Ti films were heated up to 400 ^o^C for 2 h at a ramp of 5 ^o^C•min^−1^ at atmosphere so that the resultants are of good crystallinity for high photocatalytic activity.

### Metallic nanoparticles deposition on TiO_2_ NPAs

Au/Pt NPs were deposited on TiO_2_ NPs through SILAR method with slight modification as previously reported. Briefly, the TiO_2_ NPAs substrates were successively exposed to HAuCl_4_ (or HPt_2_Cl_6_) and NaBH_4_ solutions to deposit nanocrystallites. The TiO_2_ NPAs were immersed in 0.1 mg/mL HAuCl_4_ (or 0.1 mg/mL HPt_2_Cl_6_) solution for 60 s, followed by rinsing with DI water and then immersed in NaBH_4_ solution (1 mg/mL) for another 60 s, after which the resultant was rinsed with DI water for several times. This SILAR process was repeated for several cycles until the desired quantity of metallic nanocrystallites was achieved. Here Au/Pt-TiO_2_ NPAs sample was alternately coated with Au and Pt NPs respectively for 5 times.

### Materials characterization

The morphology and structure of the samples were examined by field-emission scanning electron microscope (FE-SEM, JEOL-7001F), high-resolution transmission electron microscope (HRTEM, JEOL-2011) and Raman spectroscopy (LABRAM HR800, excitation wavelength of 633 nm), respectively. The chemical structure of the samples was analyzed by x-ray photon electron spectrometer (XPS, Perkin Elmer PHI 5300), and the binding energy was calibrated with the reference to the C1s peak centered at 284.6 eV. The optical properties of the samples were examined by a UV-Vis spectrometer (Perkin Elmer Lambda 35) in a wavelength range from 200 to 900 nm at room temperature. The photoluminescence (PL) spectra were recorded using Raman spectrometer (LabRAM ARMIS) under 325 nm excitation.

### Property Measurement

The steady sate current density and electrochemical impedance spectroscopy (EIS) measurements were carried out by an electrochemistry workstation (CHI 660D, Chenhua instrument). The nanostructured films were used as the working electrode, an Ag/AgCl electrode (saturated KCl) and Pt sheet were used as the reference and counter electrodes, respectively. The working electrode was illuminated with a 300 W Xe lamp. An ultraviolet filter was placed between the light source and the quartz cell to cut off the UV light in wavelength <420 nm. Visible light is the wavelength of light larger than 420 nm. Photocurrent densities were measured in the light on-off process with a pulse of 30 s under both UV-visible or visible light illumination (200 mW•cm^−2^) at 0.4 V bias vs Ag/AgCl electrode.

The photocatalytic activity of products was evaluated by the photodegradation of methyl orange (MO) with the light source of 300 W Xe lamp at ambient temperature. The sample on quartz substrate (15 mm** **×** **15 mm) was placed in a quartz cell containing 5 mL of MO (5 μM) solution. Prior to light irradiation, the photocatalyst was immersed into a MO solution in the dark room for 30 min to reach an adsorption/desorption equilibrium, and the Xe lamp was turned on for different time spans. After that, the concentration of MO was monitored using UV-Vis spectroscopy at 464 nm. C/C_o_ indicated percentage residue of MO at that moment. And degradation efficiency can be obtained by (1 − C/C_o_)^*^100%. PEC experiments were also performed to evaluate the photoelectrochemical property of these oxidized samples in a three-electrode cell, using the oxidized samples as the working electrode with an exposed area of 1.5 cm^2^ under light irradiation, A Pt sheet, and a Ag/AgCl electrode were used as the counter electrode and the reference electrode, respectively. Here 0.01 M KOH aqueous solution was used as the electrolyte.

## Additional Information

**How to cite this article**: Shuang, S. *et al*. Surface Plasmon Enhanced Photocatalysis of Au/Pt-decorated TiO_2_ Nanopillar Arrays. *Sci. Rep*. **6**, 26670; doi: 10.1038/srep26670 (2016).

## Supplementary Material

Supplementary Information
