# Supplementary material for: Surface Plasmon Enhanced Photocatalysis of Au/Pt-decorated TiO2 Nanopillar Arrays
Source: Sci Rep. 2016 May 24;6:26670. doi: 10.1038/srep26670 (PMC4877652; doi:10.1038/srep26670)
Supplement: Supplementary Information [file srep26670-s1.pdf]

Supplementary Information for

Surface Plasmon Enhanced Photocatalysis of Au/Pt-decorated TiO_2 Nanopillar Arrays

Shuang Shuang,[a] Ruitao Lv,[b] Zheng Xie,[a, c] and Zhengjun Zhang [b]*

a. *State Key Laboratory of New Ceramics and Fine Processing, School of Materials Science and Engineering, Tsinghua University, Beijing 100084, China*

b. *Key Laboratory of Advanced Materials (MOE), School of Materials Science and Engineering, Tsinghua University, Beijing 100084, China*

c. *High-Tech Institute of Xi'an, Xi'an 710025, China*

*Corresponding author at: S*chool of Materials Science and Engineering, Tsinghua University, Beijing 100084, China.*

E-mail: zjzhang@tsinghua.edu.cn

Supporting figure captions:

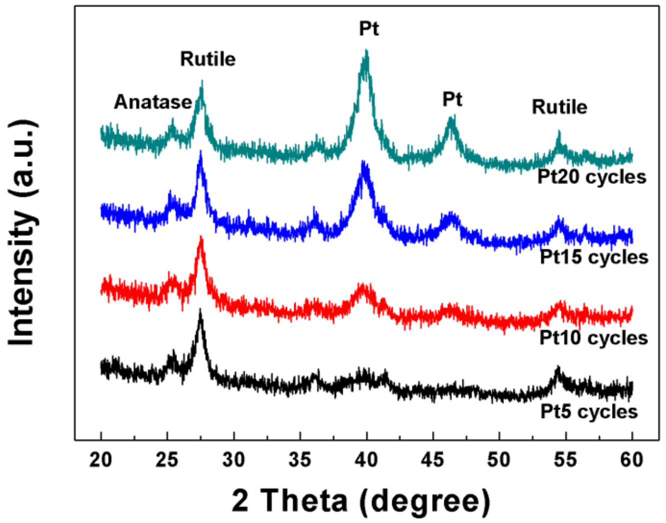

Figure S1. SEM images of Pt/TiO_2 NPAs coated with different cycles.

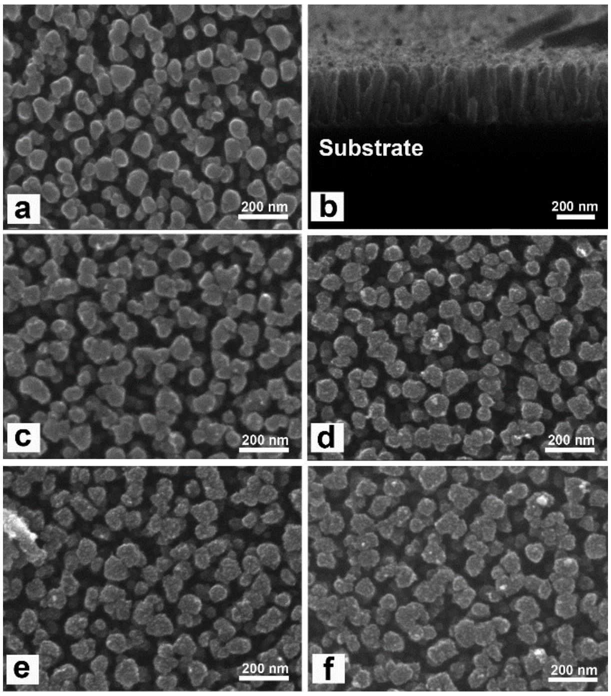

Figure S2. SEM images of the samples: (a) top view of TiO_2 NPAs; (b) sectional view of TiO_2 NPAs; Pt/TiO_2 NPAs coated with different cycles: (c) 5 cycles; (d) 10 cycles; (e) 15 cycles; (f) 20 cycles.

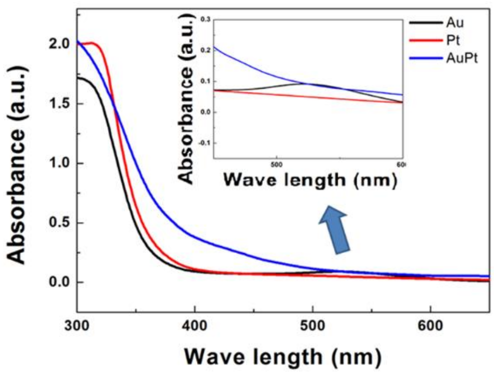

Figure S3. Absorption spectra of Au/TiO_2 NPAs coated with 10 cycles, Pt/TiO_2 NPAs coated with 10 cycles and Au/Pt-TiO_2 NPAs.

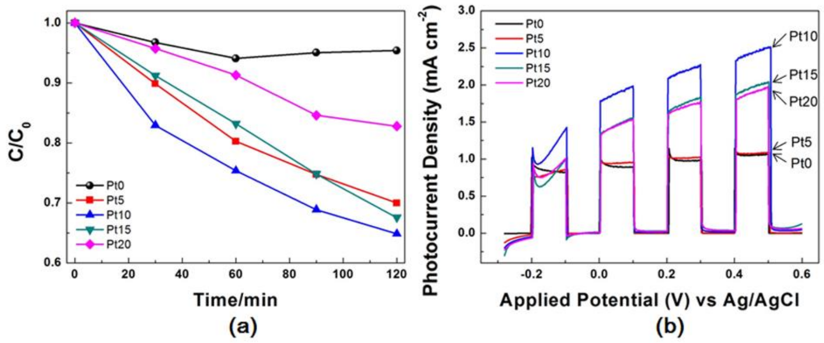

Figure S4. UV-vis light discoloration of MO (a) and current versus time measurements (b) of Pt/TiO_2 NPAs coated with different cycles.

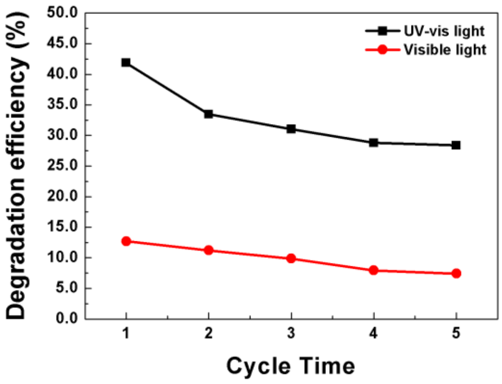

Figure S5. Cycle usage of Au/Pt-TiO_2 NPAs under both UV-vis and visible lights.

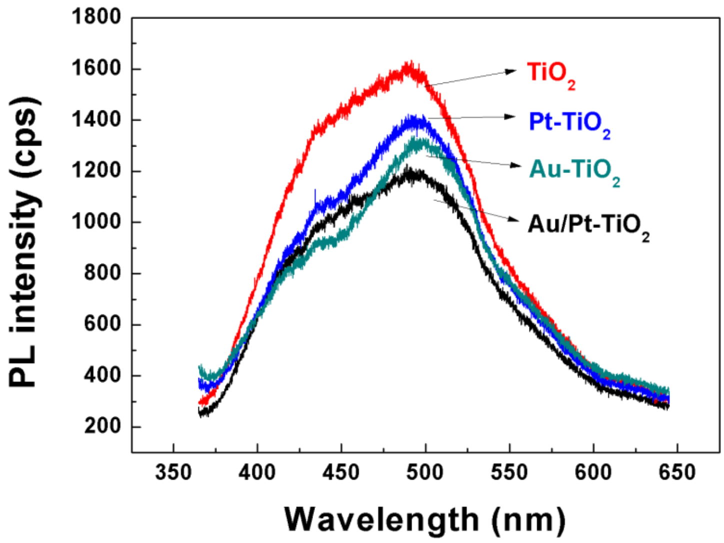

Figure S6. PL spectrum of single TiO_2 NPAs, Pt-TiO_2, Au-TiO_2 and Au/Pt/TiO_2 NPAs.
